# Regulation of Global Gene Expression in Human *Loa loa* Infection Is a Function of Chronicity

**DOI:** 10.1371/journal.pntd.0001527

**Published:** 2012-02-28

**Authors:** Cathy Steel, Sudhir Varma, Thomas B. Nutman

**Affiliations:** 1 Laboratory of Parasitic Diseases, National Institute of Allergy and Infectious Diseases, National Institutes of Health, Bethesda, Maryland, United States of America; 2 Bioinformatics and Computational Biology Branch, National Institute of Allergy and Infectious Diseases, National Institutes of Health, Bethesda, Maryland, United States of America; University of South Florida, United States of America

## Abstract

**Background:**

Human filarial infection is characterized by downregulated parasite-antigen specific T cell responses but distinct differences exist between patients with longstanding infection (endemics) and those who acquired infection through temporary residency or visits to filarial-endemic regions (expatriates).

**Methods and Findings:**

To characterize mechanisms underlying differences in T cells, analysis of global gene expression using human spotted microarrays was conducted on CD4^+^ and CD8^+^ T cells from microfilaremic *Loa loa*-infected endemic and expatriate patients. Assessment of unstimulated cells showed overexpression of genes linked to inflammation and caspase-associated cell death, particularly in endemics, and enrichment of the Th1/Th2 canonical pathway in endemic CD4^+^ cells. However, pathways within CD8^+^ unstimulated cells were most significantly enriched in both patient groups. Antigen (Ag)-driven gene expression was assessed to microfilarial Ag (MfAg) and to the nonparasite Ag streptolysin O (SLO). For MfAg-driven cells, the number of genes differing significantly from unstimulated cells was greater in endemics compared to expatriates (p<0.0001). Functional analysis showed a differential increase in genes associated with NFkB (both groups) and caspase activation (endemics). While the expatriate response to MfAg was primarily a CD4^+^ pro-inflammatory one, the endemic response included CD4^+^ and CD8^+^ cells and was linked to insulin signaling, histone complexes, and ubiquitination. Unlike the enrichment of canonical pathways in CD8^+^ unstimulated cells, both groups showed pathway enrichment in CD4^+^ cells to MfAg. Contrasting with the divergent responses to MfAg seen between endemics and expatriates, the CD4^+^ response to SLO was similar; however, CD8^+^ cells differed strongly in the nature and numbers (156 [endemics] vs 36 [expatriates]) of genes with differential expression.

**Conclusions:**

These data suggest several important pathways are responsible for the different outcomes seen among filarial-infected patients with varying levels of chronicity and imply an important role for CD8^+^ cells in some of the global changes seen with lifelong exposure.

## Author Summary

Infection with the filarial parasite *Loa loa* causes a parasite-specific downregulation of T cell responses. However, differences exist (clinical and immunologic) between patients born and living in filarial endemic regions (endemics) and those who become infected during travel or short-term residency (expatriates). T cell responses are more depressed in endemics while expatriates have more clinical “allergic-type” symptoms. In this study, we showed that these differences reflect transcriptional differences within the T cell compartment. Using microarrays, we examined global gene expression in both CD4^+^ and CD8^+^ T cells of microfilaremic endemic and expatriate patients and found differences not only *ex vivo*, but also to parasite and, for CD8^+^ cells, to nonparasite antigens. Functional analysis showed that endemic patients expressed genes linked to inflammatory disease and caspase associated cell death at homeostasis while expatriates tended to have a more activation-induced gene profile at homeostasis and a CD4^+^ inflammatory response to parasite antigen. Patient groups were similar in their CD4^+^ response to nonparasite antigen but strongly differed in their CD8^+^ responses, demonstrating the potential global ramifications of chronic, longstanding infection. Our study describes potential transcriptional mechanisms for the variability seen in patients with different levels of exposure to and chronicity of filarial infection.

## Introduction

Infection with the pathogenic filariae, *Loa loa*, *Brugia malayi*, *Wuchereria bancrofti*, and *Onchocerca volvulus*, causes an enormous disease burden throughout tropical and sub-tropical regions of the world. Interestingly, however, the clinical manifestations of infection are often markedly different in those with lifelong exposure (i.e. those born in filarial-endemic regions) and those that acquire infection later in life through travel to or temporary residence in a filarial-endemic area [1], [2]. Indeed, filarial infections are less likely to be subclinical in expatriates [3] or transmigrants [4] compared to those with lifelong exposure. Expatriates with loiasis, for example, are more likely to have Calabar swellings and other “allergic” phenomena – such as marked peripheral blood eosinophilia, elevated IgE levels, and urticaria – than is seen in the more chronically infected patients born and raised in endemic areas [5].

With availability of more sensitive assays for the definitive diagnosis of filarial infections, it is now known that infection occurs at much earlier ages than once believed [6], although relatively intense exposure to the vectors that transmit these infections is typically required for acquisition of infection. However, expatriates who acquire infection are not subject to many of the environmental and familial factors that affect those born in endemic regions, the most notable being the alteration of immune responses specific for filarial antigens that occurs early in life [7], [8], [9], and that can persist long-term (decades) [10] as a consequence of *in utero* exposure to filarial antigens. Moreover, polyparasitism is much more frequent among patients from filarial-endemic regions than in expatriates. That individuals living in an endemic area are exposed continually to the parasite, irrespective of the infection status, is evidenced by the Ag-specific antibody responses seen among filarial-uninfected endemic individuals [11], [12]. Indeed, both susceptibility to infection and the nature of the immune response has a significant genetic component in helminth- and filarial-endemic populations [13], [14], [15], [16].

Several studies have also demonstrated differences in immune responses to filarial antigens among filarial-infected travelers (expatriates) and those from filarial-endemic regions [1], [2]. Filarial-infected individuals from endemic countries, while having increased antifilarial IgG4 antibodies [17], have more profoundly diminished parasite-specific T cell responses [12], [18] than those seen in expatriates [1]. This parasite-specific hyporesponsiveness is reflected not only in diminished proliferative and cytokine responses [12], [18], [19], but also in the increased expression of molecules (e.g. CTLA-4, PD-1) known to inhibit T cell responses [20], [21]. In addition, filarial Ags and live filarial parasites have themselves been shown to induce proliferative defects [22], apoptosis of T cells [23], and impairment of antigen presenting cell number and function [24], [25], [26], that cumulatively may alter T cell responses.

A number of studies have directly examined specific (or candidate) pathways in the cells of filarial-infected [24], [25] individuals. To examine more globally the differences in responsiveness to filarial infections between persons with relatively newly acquired infection and those with lifelong exposure and to evaluate more comprehensively the T cell responses (both CD4^+^ and CD8^+^) seen in these two groups, we utilized spotted, human microarrays and RNA from either CD4^+^ or CD8^+^ T cells (*ex vivo*) and in response to filarial and nonfilarial antigens. Our findings demonstrate a striking difference in gene expression between endemic and expatriate patients with the same filarial infection and demonstrate that these differences manifest not only in T cells *ex vivo* but also in response to both parasite and nonparasite Ag.

## Materials and Methods

### Patient Groups and *in vitro* Cell Culture

All patients were seen under a protocol (NCT00001230) that was approved by the Institutional Review Board of the National Institute of Allergy and Infectious Diseases, National Institutes of Health (NIH), and informed written consent was obtained from all subjects. Three *Loa loa*-infected patients who had lived most or all of their lives in a region endemic for loiasis and 3 expatriate *L. loa*-infected individuals were chosen for study (Table 1). All patients were examined at the NIH, and all had demonstrable microfilariae in their circulation at midday. None of the patients tested positive for HIV. One expatriate patient (but no others) had intestinal parasites as well. PBMCs from all patients were collected prior to any treatment, cryopreserved using standard techniques and stored in liquid nitrogen until used. For this study, cryopreserved PBMCs were thawed and then layered over Ficoll/diatrizoate (MP Biomedical, LLC, Solon, OH) to separate viable cells from dead cells. Cells at the interface, were collected, washed, and counted (>98% viable by trypan blue exclusion). Cells were cultured in 12-well plates in RPMI-1640 (Invitrogen, Carlsbad, CA) supplemented with 10% fetal calf serum (Gemini BioProducts, Woodland, CA) at 10×10^6^ cells/well in the absence (media alone) or presence of a PBS extract of microfilariae (MfAg, 10 mg/ml) or with a non-parasite control Ag Streptolysin O (SLO, 1∶100 final concentration; Difco, Detroit, MI). Wells with media alone were run for each antigen and for each cell type (i.e. a total of 4 wells with media alone were cultured for each patient).

**Table 1:** Patient Population.

Patient	Sex	Age at 1^st^ NIH Visit	Age at Initial Exposure	Country Loa Acquired	Mf/mL	History of Calabar Swellings	Eyeworms	Other Parasites Identified
Endemic 1	M	25	N/A	Cameroon	650	No	Yes	None
Endemic 2	F	34	N/A	Nigeria	2020	Yes	No	None
Endemic 3	M	41	N/A	Gabon	5540	No	No	None
Expatriate 1	F	25	25	Cameroon; CAR; DRC	753	Yes	Yes	Intestinal Parasites*
Expatriate 2	F	26	23–25	Gabon	8260	Yes	Yes	None
Expatriate 3	F	25	23	Cameroon	4000	Yes	No	None

*Expatriate 1 infected with *A. lumbricoides, G. lamblia, T. trichiura, and I. belli*.

### CD4^+^/CD8^+^ T cell Selection and RNA Preparation

Following a 16 hr. incubation, PBMCs were harvested and washed in PBS/0.1% BSA/2 mM EDTA. Primary selection for CD3^+^ cells was accomplished using the Dynal negative T cell isolation kit II (Invitrogen, Carlsbad, CA) known to retain activated T cells. After negative selection, positive selection for either CD4^+^ or CD8^+^ T cells was accomplished using Dynal beads, giving a >99% pure population of each cell type as determined by flow cytometry.

After selection, cells were immediately homogenized in 1 ml Trizol (Invitrogen) followed by phase separation with chloroform. Following the addition of 70% ethanol to the RNA-containing aqueous phase, RNA was further purified using the RNeasy Kit (Qiagen, Valencia, CA) following the manufacturer's instructions. RNA concentrations were analyzed on a NanoDrop spectrophotometer (NanoDrop Technologies, Wilmington, DE) and quality was assessed using the Agilent 2100 Bioanalyzer (Agilent Technologies, Santa Clara, CA).

### RNA RT-PCR, Amplification and Labeling

From a starting template of 40 ng RNA, cDNA was synthesized by reverse transcription, then amplified and labeled using the Ovation Aminoallyl RNA Amplification and Labeling System (NuGen Technologies, San Carlos, CA) following the manufacturer's instructions. Technical replicates were done for each sample. The resulting aminoallyl labeled cDNA was purified using QIAquick columns (Qiagen) and cDNA concentrations were measured.

### Microarray Hybridization

For each patient sample (CD4^+^ and CD8^+^ cells), replicate cDNA from Ag-driven and media samples were processed concurrently for microarray analysis; because each of the two Ags were run in duplicate, cDNA from all four media samples was used in the analyis of unstimulated cells. For hybridization, aminoallyl cDNA was first labeled with cyanine (Cy) dyes (Amersham, Piscatawey, NJ) using Cy3 dye for antigen-driven samples and Cy5 dye for samples from media alone. Following purification on QIAquick columns, corresponding Ag and media Cy3 and Cy5 labeled samples were combined, concentrated, and then hybridized to human spotted arrays (NIAID - Hsbb; Platform GPL1054) printed by the NIAID/Microarray Research Facility. The probe set for the microarrays was based on 70 mer oligonucleotides from the Human Genome Oligo Set V2.0 (Qiagen). Each array contained 21,531 oligonucleotides. Microarray chips were imaged with a GenePix 4000B fluorescent scanner (Molecular Devices, Sunnydale, CA). Data has been deposited in the National Center for Biotechnical Information (NCBI) Gene Expression Omnibus at http://www.ncbi.nlm.nih.gov/geo/query/acc.cgi?token=vnitzmsyymoigly&acc=GSE31894, GEO accession number (GSE31894). Processed data can be accessed by using the “Series Matrix File(s)” link.

### Microarray Data Analysis

#### Data import and normalization

Microarray data was imported from GenePix Results (GPR) files into Partek Genomics Suite with the option to average duplicate genes using the median. Intensity values less than 100 were given a threshold of 100, followed by conversion to the log base 2; each sample was then centered about the median. Subsequently, genes with a mean <7 or a standard deviation <0.7 were removed and median normalization was repeated for each sample.

#### Media treated (unstimulated) samples

To establish the differential expression between the unstimulated cells from expatriate and endemic patients, an ANOVA model was created with the following factors: 1) Fixed effects - Patient-type, Cell-type, and Stimulation; 2) Random effects – Print ID and Patient ID; and 3) Interaction effects – Patient-type*Cell-type, Cell-type*Stimulation, Stimulation*Patient-type, and Patient-type*Cell-type*Stimulation. Using this ANOVA model, comparisons were made between endemic and expatriate CD4^+^ and CD8^+^ T cells. The p-values for the differential expression of each gene were adjusted for multiple comparisons using the Benjamini Hochberg False Discovery Rate (FDR) correction [27] and the fold change between the two patient groups was then calculated as expression by endemic patients/expression by expatriate patients. Genes found to have significant expression (p<0.01) were annotated and placed into functional networks using Ingenuity Pathway Analysis® (http://www.ingenuity.com). A particular function was considered significant if the number of genes within that function was considered greater than that expected by chance (p<0.05) using the Bonferroni correction for multiple comparisons.

Gene Set Enrichment Analysis (GSEA; http://www.broadinstitute.org/gsea/index.jsp; [28], [29]) was used to determine biological pathways that may be altered in endemic patients as compared with expatriates. Genes were ranked based on the formula (−log base 10 [p-value] * sign [fold change]). By this analysis, the negative log of the p-value for a given gene increases with increasing statistical significance of the gene (i.e. decreasing p-value). However, since the p-value does not give an indication of the direction of fold change, the negative log of the p-value was multiplied by the sign of the fold change between endemic and expatriate samples for a given gene. Functional groups of genes whose ranks were significantly lower or higher than would be expected by chance were derived in GSEA; a group of genes with common function and high ranking was evidence that the function was up-or-down-regulated in one patient group over the other. The Canonical Pathways gene sets (version 2.5) used in GSEA were obtained from the Molecular Signatures Database using the Curated Gene Sets category (GSEA-MSigDB Canonical Pathways; available at http://www.broadinstitute.org/gsea/msigdb/genesets.jsp?collection=CP).

To determine whether certain functions were enriched, a clustering analysis was done of genes that showed significant differential expression between endemic and expatriate patients with a FDR<0.1. Genes were divided into several clusters according to consistent positive or negative regulation across the samples and the Fisher Exact test was used to calculate the significance of the number of genes belonging to a particular Canonical Pathway. A small p-value denotes that for a given pathway, the number of genes belonging to that pathway within a cluster was greater than would be expected by chance.

#### Antigen-treated (stimulated) samples

For Mf- and SLO-stimulated samples, genes that were significantly either over- or under-expressed in comparison with paired unstimulated samples were analyzed; the ratio of stimulated to unstimulated expression was then calculated as the fold change for a particular gene. Since there were very few genes that showed significant differential expression between Ag-driven and unstimulated samples using the Benjamini Hochberg FDR correction ([27]; FDR<0.1), a more stringent p-value (p<0.001) was used for determination of significance without the correction for multiple comparisons. Genes found to have significant expression in antigen-driven cells with respect to unstimulated cells in this manner were then annotated and placed into functional networks using Ingenuity Pathway Analysis®. Functional gene sets that were enriched overall with small p-values were detected by means of GSEA using the Canonical Pathways gene sets similar to that done for unstimulated data.

### Real-Time Quantitative RT-PCR (Taqman™)

To confirm the quality of the microarray findings, identical RNA was used for comparison to quantitative real-time reverse transcription (RT-)PCR (Taqman™) on a representative selection of differentially expressed genes in unstimulated cells (10 for CD4^+^ cells, 7 for CD8^+^ cells). Pre-developed assay reagents, primers, and probes were obtained from Applied Biosystems (Branchburg, New Jersey) and run per the manufacturer's instructions using an ABI 7900 Real-Time PCR System. All samples were run in triplicate and normalized to their own 18S ribosomal RNA. Gene expression for each patient was calculated as the antilog of ([1/ΔCT]×100) where ΔCT = cycle threshold (CT) of the test gene minus the CT of the 18S ribosomal gene.

To establish if there was a correlation between the expression of a given gene using RT-PCR and the expression determined by microarray, the 3 patient values for each gene within each group were averaged for both techniques and then plotted against each other. Correlation values were assessed by the Spearman Rank Test using GraphPad Prism 5.0.

### Other Statistical Analyses

To determine differences between numbers of genes expressed between the two patient groups, a statistical test for one proportion was done using the Normal approximation. For each category, the number of genes assigned to that category in the two classes (endemic and expatriate) was compared to each other. Assuming a null proportion of 0.5 (i.e. that there is no difference in the number of genes of that category for the two classes), p-values were calculated for deviation from 0.5 using a Normal approximation.

## Results

### RNA Expression in Unstimulated Cells

**Figure 1:**
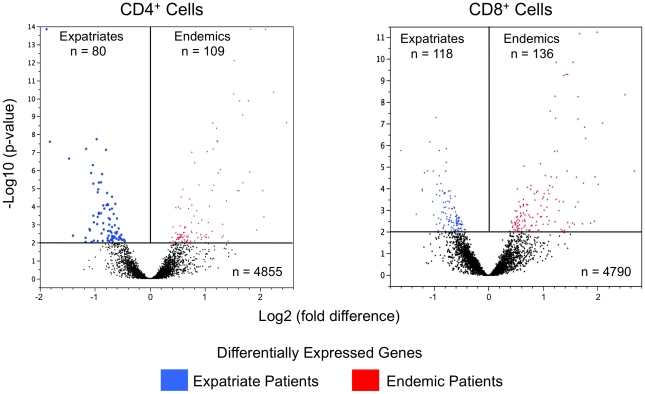
Volcano plots of differentially expressed genes. Volcano plots of differentially expressed genes (p<0.01) in the unstimulated CD4^+^ (left panel) and CD8^+^ (right panel) T cells of filarial-infected expatriate (blue dots) and endemic (red dots) patients. Genes not significantly different are symbolized by black dots with each dot denoting a single gene. The x-axis is the fold difference (log 2) between patient groups and the y-axis represents the log10 of the p-value.

**Figure 2:**
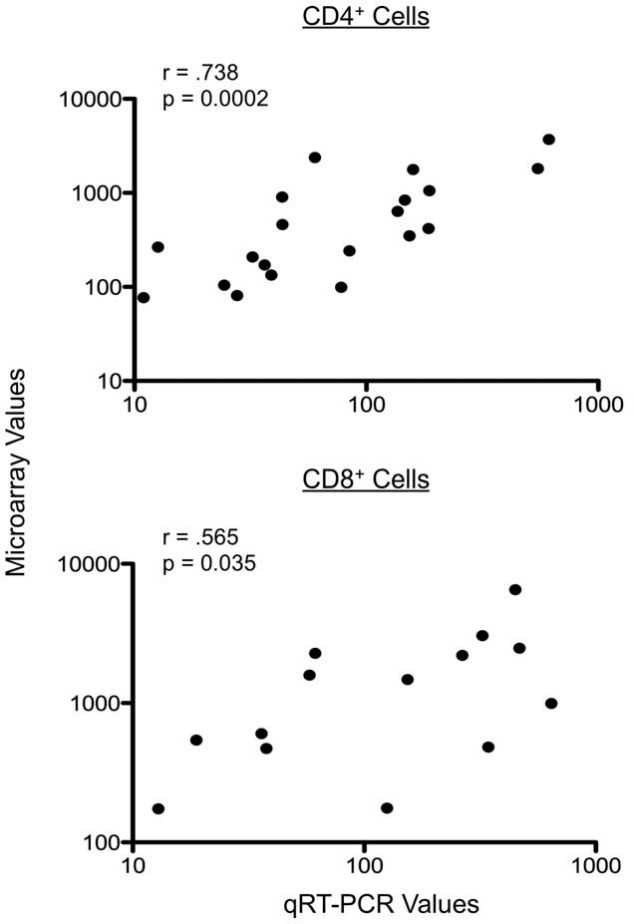
Quantitative RT-PCR and microarray correlation. Correlation between Taqman™ qRT-PCR values and microarray expression values for 10 genes in CD4^+^ cells (top panel) and 7 genes in CD8^+^ cells (bottom panel). Each point illustrates the average value of the 3 individuals within each patient group for qRT-PCR (x-axis) vs microarray (y-axis) for the same sample. Quantitative RT-PCR values were calculated as the antilog of ([1/ΔCT]×100) where ΔCT = cycle threshold (CT) of the test gene minus the CT of the 18S ribosomal gene; microarray values are the antilog of the intensity values imported from GenePix. The Spearman Rank Correlation was used to calculate r- and p-values. Data associated with these plots are shown in Table S1.

Among the 21,531 oligonucleotides on the arrays, expression by CD4^+^ and/or CD8^+^ T cells could be detected in ∼23% (n = 5044). As can be seen in Figure 1, those subjects with lifelong exposure (hereafter referred to as endemics) had slightly greater numbers of differentially-expressed genes than did the expatriates (109 vs 80 for CD4^+^ cells; 136 vs 118 for CD8^+^ cells; at p<0.01, corrected for FDR). Seventeen of these differentially expressed genes were independently validated using RT-PCR, as shown in Figure 2 and Table S1 in which the expression values from the microarray data were correlated with those data derived from the RT-PCR (p = 0.0002 for CD4^+^ cells [10 genes], and p = 0.035 for CD8^+^ cells [7 genes]; Figure 2).

**Figure 3:**
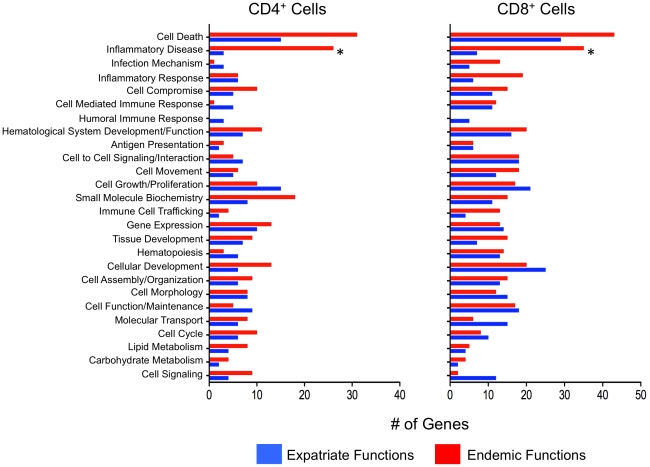
Functional analysis of genes in unstimulated cells. Number of genes associated with specific cellular functions in the unstimulated CD4^+^ (left panel) and CD8^+^ (right panel) T cells of *Loa loa* infected endemic (red bars) and expatriate (blue bars) patients. Annotation for cell functions was extracted from www.ingenuity.com using genes that were differentially expressed at p<0.01 and adjusted for multiple comparisons using the Benjamini Hochberg False Discovery Rate (FDR) correction.

When the genes expressed differentially between patient groups were analyzed further, the differences between endemic and expatriate patients could be inferred through functional assessments of these gene sets (Figure 3). For CD4^+^ cells, endemic patients over-expressed a significantly greater number of genes related to inflammatory disease (26 vs 3 for expatriates; p<0.0001). Cell death-associated genes were also over-represented in endemic patients (31 genes vs 15 in expatriates) although the difference did not reach statistical significance when corrected for multiple comparisons (p = 0.09). Although also not statistically different, expatriates had an overrepresentation of genes associated with transcriptional activity, cell function/maintenance and cell growth/proliferation.

Overall, the number of genes that were differentially expressed by either patient group was greater in the CD8^+^ than in the CD4^+^ T cells. Similar to the findings for CD4^+^ cells, the CD8^+^ cells in endemic patients showed an expansion in the number of genes involved in pathways associated with “inflammatory disease” (35 genes in endemics vs 7 in expatriates, p = 0.0003; Figure 3) and with cell death (43 vs 29; p>0.05). In contrast, the number of genes implicated in cell signaling (12 vs 2) and molecular transport (16 vs 6), though not significantly different, were overrepresented in the CD8^+^ cells of expatriates.

**Figure 4:**
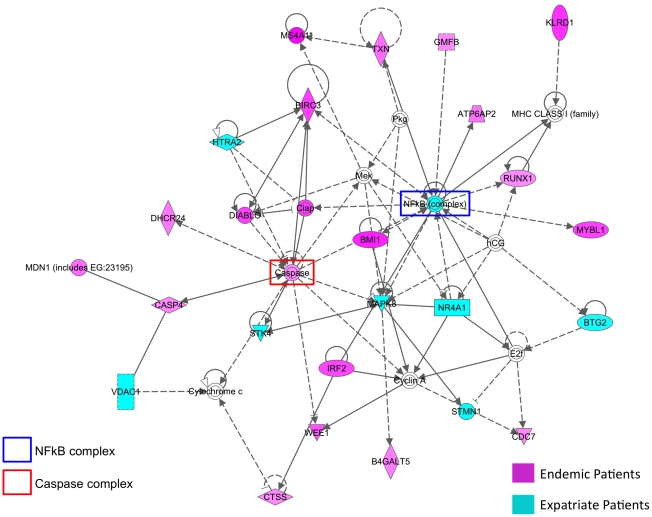
Cell death network in unstimulated cells. Network of differentially expressed molecules associated with cell death (www.ingenuity.com) in the unstimulated CD4^+^ and CD8^+^ T cells of endemic (in red) and expatriate (in blue) patients. Genes were differentially expressed at p<0.01, adjusted for multiple comparisons using the Benjamini Hochberg False Discovery Rate (FDR) correction. Lines represent direct (solid lines) and indirect (dashed lines) relationships between molecules and molecules within double circles represent complexes of genes.

Both groups had a large number of differentially expressed genes related to cell death (Figure 4). As can be seen in this representative network, the endemic patients upregulated many of the genes involved in caspase activation whereas the expatriates were more likely to have relatively upregulated activation of the NFkB complex, including MAPK8, part of the p38 MAPK activation complex. An additional network associated with IFN-γ, IFN-α, and IL-2 was also seen for the CD4^+^ cells of expatriates (data not shown). Furthermore, expatriates showed a relative upregulation of several pro-apoptotic genes including PDCD4 (programmed cell death 4; CD4^+^ and CD8^+^ T cells), HTRA2 (HtrA serine peptidase 2; CD8^+^ T cells), and STK4 (serine/threonine kinase 4 [MST1]; CD4^+^ cells) and some involved in anergy induction (DGKA (diacylglycerol kinase-alpha; CD8^+^ cells; data not shown).

While pro-inflammatory and activation-associated networks were also seen in endemic patients, represented by genes that included KLRD1, CCL4, CXCR4, JAK2 and HLA-DRA [Figure 4, Figure S1, and Figure S2]), there was, in addition, a differential increase in the expression of several immunoregulatory molecules including IRF2 (interferon regulatory factor 2; CD4^+^ and CD8^+^ cells), TIMP2 (TIMP metallopeptidase inhibitor 2; CD4^+^ cells), PTGDR (prostaglandin D2 receptor; CD8^+^ cells), and MAF (musculoaponeurotic fibrosarcoma oncogene homolog; CD8^+^ cells). More importantly, the endemic patients differed in the number of genes directly linked to the increase (DIABLO, CASP4, MS4A1, GNLY) or inhibition (BIRC3, DHCR24) of caspase-dependent (Figure 4) or caspase-independent (CD99; data not shown) apoptosis, suggesting there was a fine balance between pro- and anti-apoptotic molecules in the baseline CD4^+^ and CD8^+^ cell response to filarial infection *in vivo*. In addition to the prominent cell death/inflammatory networks discussed above, CD8^+^ T cells of endemic patients also demonstrated a differential increase in molecules coding for proteins with cytotoxic effector function, among them the killer cell lectin-like receptor molecules KLRB1 and KLRD1, granzyme A (GZMA) and granulysin (GNLY).

Hierarchical clustering and Gene Set Enrichment Analysis (GSEA) was utilized to determine whether patient groups could be distinguished by their canonical pathway profiles. Analysis of CD4^+^ cells (Figure S1), showed significant enrichment (enrichment of 56%; corrected p<0.01) in only the Th1/Th2 pathway among the endemic patients compared to the expatriate subjects. In marked contrast, several pathways were significantly and differentially enriched in CD8^+^ T cells from both patient groups (Figure S2). Among the canonical pathways in which there was enrichment in endemic patients were the IL-5 (150%; p<0.1) and eosinophil (75%; p<0.1) pathways. Two pathways enriched by ∼150% with respect to expatriate patients were the Asbcell and BBcell pathways, both which are involved in T cell-B cell interactions. The most highly enriched canonical pathways in expatriates included the PAC1 receptor pathway (>150%; p<0.01) and the WNT Ca2 cyclic GMP pathway (95%; p<0.01), as well as the complement/coagulation and granulocyte cell survival pathways.

### Antigen Driven RNA Expression

**Figure 5:**
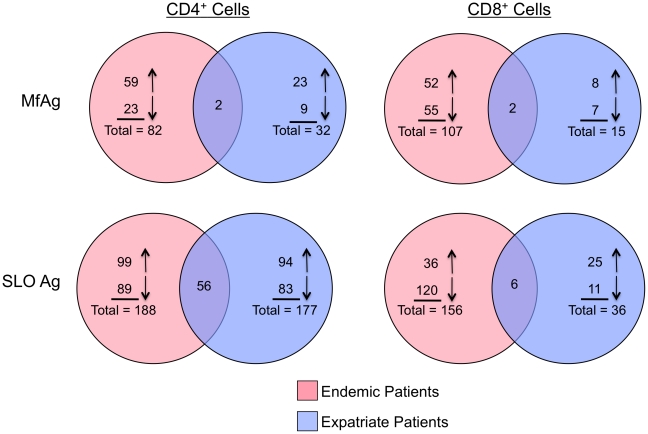
Venn diagrams illustrating the number of Ag-driven genes. Venn diagrams representing the number of genes either upregulated (upward facing arrows) or downregulated (downward facing arrows) to parasite (microfilarial Ag; MfAg, top panels) and nonparasite (streptolysin O; SLO, bottom panels) antigens in endemic (pink circles) and expatriate (blue circles) filarial-infected patients. CD4^+^ T cells are shown in the left panels and CD8^+^ T cells are in the right panels. Numbers represent genes that were significantly up-or down-regulated (p<0.001) in comparison to unstimulated cells.

Figure 5 shows the number of genes that were either upregulated or downregulated in response to filarial MfAg or to the nonparasite Ag SLO compared to unstimulated cells (based on a paired p-value<0.001). As can be seen, the response to either Ag (in general) was different between the two patient groups. Interestingly, in response to MfAg, only the chemokine ligand CCL3 and tubulin folding cofactor A (TBCA) in CD4^+^ cells and glyceraldehyde-3-phosphate dehydrogenase (GAPDH) and TBCA in CD8^+^ cells were upregulated in common between the patient groups. In addition, there were relatively few (n = 21) genes that were regulated similarly to both antigens (MfAg and SLO) inclusive of both groups of patients and both cell types, suggesting that the nature of the antigen (and presumably the method of sensitization) plays a major role in shaping the antigen-specific T cell response. In general, the more chronically infected patients (endemics) had a greater number of antigen-induced differentially regulated genes than did the expatriates; however, the most striking difference was in the number of genes differentially expressed by CD8^+^ cells (107 vs 15, p<0.0001; Fig. 5).

Genes that were significantly up- or down-regulated (p<0.001) in response to MfAg compared to their expression in unstimulated cells were assessed through pathway analysis. For CD4^+^ T cells, these functions included cell death, cell assembly and organization, cell development, and cell function and maintenance in endemic patients, while in expatriates the most prominent functions were cell assembly and organization, cell function and maintenance, and cell movement (data not shown). Similarly, the principal functions associated with CD8^+^ T cells included cell growth and proliferation, cell development, and cell death in endemic patients, and cell death, inflammatory disease, and gene expression in expatriates.

**Figure 6:**
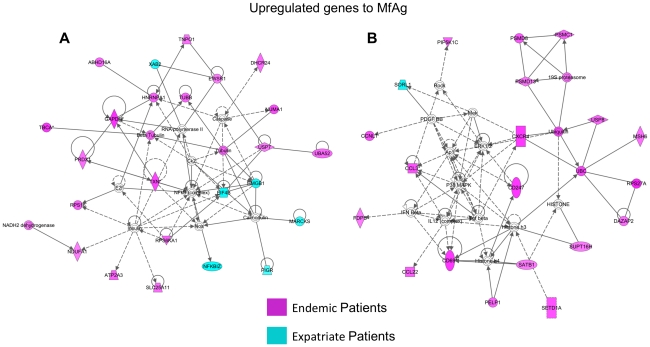
Networks of microfilarial Ag-driven upregulated genes. Two major networks (Figures A and B; www.ingenuity.com) of differentially expressed molecules from either CD4^+^ or CD8^+^ T cells that were significantly upregulated (p<0.001) to MfAg in endemic (in red) and expatriate (in blue) patients. Lines represent direct (solid lines) and indirect (dashed lines) relationships between molecules, and molecules within double circles represent complexes of genes.

To establish an overall profile of genes upregulated in loiasis patients in response to MfAg, composite networks were identified (representative networks shown in Figure 6). Figure 6A illustrates that the majority of genes with altered expression from that seen in unstimulated cells were those associated with the NFkB (both patient groups) and Caspase (endemic group) complexes. For the endemic patients, however, an increase in the expression of genes linked to insulin and insulin signaling in both CD4^+^ and CD8^+^ T cells (Figure 6A and data not shown) could be seen to be induced by MfAg. A second composite network (Figure 6B) consisted chiefly of those genes upregulated in the T cells from endemic patients. This analysis clearly identified several molecules involved in activation, cell trafficking, and Ag presentation, including CD69 in CD4^+^ and CD8^+^ cells, CXCR4 and CCL3 (MIP1α) in CD4^+^ cells, and CD247 (TCRζ) and CCL22 in CD8^+^ cells. Further analysis of genes upregulated in endemic patients showed molecules associated with histone complexes (such as histone h3) in both CD4^+^ and CD8^+^ cells (Figure 6B), with the protein kinases AKT, P38MAPK, JNK, and ERK1/2 in CD4^+^ cells (Figure 6B and data not shown), and with ubiquitin related molecules in CD8^+^ cells (Figure 6B).

As indicated in Figure 5, because of the differences in numbers of genes with increased expression to MfAg, the contribution by expatriates to the overall composite profile was far less than that by the endemic patients. Moreover, when networks were examined by cell type, it was apparent that the majority of the response to MfAg in expatriates was associated with the CD4^+^ T cells (Figure 5 and data not shown), differing from endemic patients in which responses were seen in both CD4^+^ and CD8^+^ cells. The most clear-cut contribution by expatriates to the overall response (Figure 6A) was an upregulation of genes associated with the calcium-binding protein calmodulin. Further analysis of individual networks derived from the CD4^+^ cells of expatriates determined that their response to MfAg was largely a pro-inflammatory one, comprised of genes either directly or indirectly associated with cytokines (IL-6 and IL-12), chemokines (CCL3 [MIP1α]), or with the high mobility group box 1 (HMGB1) molecule, known to be involved in the inflammatory response to antigenic stimuli (data not shown).

**Figure 7:**
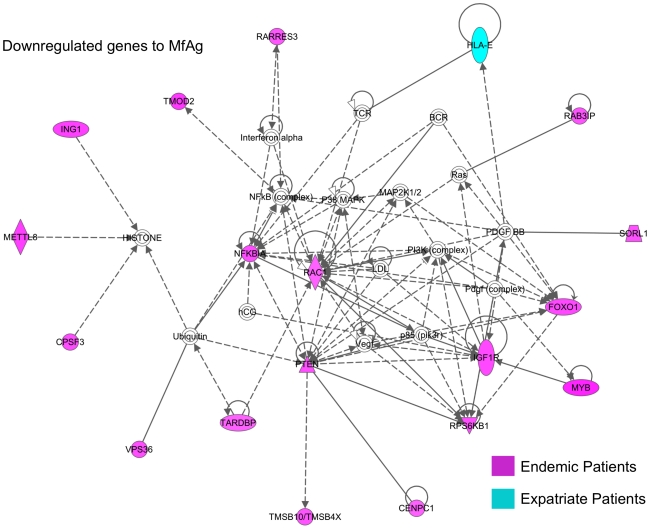
Network of microfilarial Ag-driven downregulated genes. Major network (www.ingenuity.com) of differentially expressed molecules that were significantly downregulated (p<0.001) to MfAg in either CD4^+^ or CD8^+^ T cells of endemic (in red) and expatriate (in blue) patients. Lines represent direct (solid lines) and indirect (dashed lines) relationships between molecules and molecules within double circles represent complexes of genes.

To establish the profile of downregulated genes in response to MfAg in loiasis patients, composite networks were again created (representative network shown in Figure 7). In the CD4^+^ cells of endemic patients, insulin-like growth factor I receptor (IGF1R) and RARRES3, a member of the suppressive retinoid family (Figure 7 and data not shown) were markedly downregulated in response to MfAg, while in CD8^+^ T cells, members of the GTPase RAS superfamily of GTP binding proteins (RAC1) and the reticulon family (RTN4), as well as inhibitors of the NFkB (NFKB1A) and AKT (PTEN) protein kinase complexes were decreased (Figure 7 and data not shown). Further analysis of CD8^+^ networks showed that molecules either directly or indirectly associated with TNF, IL1B, and TGFB1 were also downregulated in endemic patients relative to their expression in media (data not shown). The few molecules downregulated in expatriate T cells included FURIN (in CD4^+^ cells) and HLA-E (CD8^+^ cells; Figure 7).

Genes that were significantly up- or down-regulated to MfAg with respect to unstimulated cells were also examined by GSEA. Pathways associated with two chemokine receptors (CCR5 and CXCR4 [40% and 35% enrichment respectively] and for the transcriptional corepressor PELP1 (60%) were enriched (and upregulated) in MfAg-stimulated cells from endemic patients (Figure S3). MfAg-driven downregulated pathways associated with the pro-apoptotic molecule BAD (52%) as well as ERK (25%) were also enriched in endemic patients. Interestingly, there were several canonical pathways associated with the insulin growth factor 1 molecule that were decreased in response to MfAg, including the IGF1, Longevity, and IGF1 MTOR pathways (52%, 45%, and 23% enrichment respectively).

In contrast to what was seen in GSEA analysis for endemic patient cells, expatriate patients' cells in response to MfAg had enriched pathways associated with the innate and adaptive inflammatory response (Figure S4). These included the pathways for Notch signaling (most highly enriched at >500%), erythrocyte differentiation (180%), NKT activation (70%), and Toll-Like Receptor Signaling (25%). The DNA fragment pathway (>500%) was the major pathway downregulated in expatriates suggesting antigen-induced anti-apoptotic mechanisms.

In marked contrast to the observations of responses to MfAg were the findings to the nonparasite Ag SLO. Most noticeable was the relative commonality in the responses seen in CD4^+^ T cells from both the endemic and expatriate patients (Figure 5) with both the numbers of up- and down-regulated genes and functional pathways associated with transcriptional activity (cell death, cell growth and proliferation, and gene expression) being similar. Of note, the response to SLO in both groups consisted of increased expression of several chemokine ligands (including CCL20, CCL4 and CCL8), interferon regulatory factors, genes associated with inflammatory cytokines, and importantly, the signaling molecule STAT1, necessary for the production of interferons (data not shown).

The response in CD8^+^ T cells to SLO, however, was dramatically different between the endemic patients and the expatriates with the endemic population having many more genes altered (n = 156 vs 36). Moreover, 77% (120/156) of these genes were downregulated compared to Ag-unstimulated cells (Figure 5) with many being either indirectly or directly associated with NFkB (data not shown). Of particular interest was the observation that only endemic CD8^+^ T cells failed to upregulate STAT1 in response to SLO (data not shown).

## Discussion

For some time now, it has been known that differences in clinical manifestations exist between filarial-infected patients with lifelong exposure and those with significantly less exposure (travelers/temporary residents; [1], [2], [5]). It has been felt that these disparities may reflect differences in immunologic responsiveness to the parasite in these patients. To address specifically the concept that chronicity of infection results in differences in the nature of immune responses to that infection, we examined the global gene expression of both CD4^+^ and CD8^+^ T cells from individuals who differed primarily in the length of time infected with the blood-borne filarial parasite, *Loa loa* (Table 1). Our data suggest that, while the expression of the majority of genes (>5,000) examined by microarray was similar between the two groups, there were significant differences in the T cell responses *ex vivo* as well as in response to parasite antigen and even to a bystander antigen. Previous work has demonstrated that cells of filarial-infected endemic patients have markedly diminished parasite-specific T cell responses when compared to filarial-infected expatriate patients and even to uninfected endemic individuals [1], [12]. In a study of transmigrants to an *O. volvulus*-endemic area from a non-endemic region, it was shown that recent infection was associated with vigorous parasite-specific proliferative and cytokine responses that differed in comparison to the diminished responses seen in the chronically infected patients [30]. Similar findings have been seen in patients with acute or subacute schistosomiasis infection who had higher parasite-specific proliferative responses than did those with longstanding, chronic infection [31]. In addition, filarial-infected patients from endemic regions of the world cured of filarial infection following treatment continue to show diminished T cell responses to filarial antigens [32] while expatriate patients cured of infection (and not re-exposed) recover many of their Ag-specific T cell responses [33].

A major finding in this study was the importance of the inflammatory and cell death networks in the *ex vivo* (unstimulated) cells of filarial-infected patients in both patient groups though individual genes within these networks segregated by patient group. For example, within cell death networks the expatriates were more likely to express genes associated with activation induced cell death whereas the endemic patients expressed genes associated with apoptosis. That increased cellular activation, cell death, and inhibition of cell death is occurring at a steady-state, suggests that under conditions of long-term Ag stimulation, a balance between pro- and anti-apoptotic transcriptional events (e.g. DIABLO and BIRC3; [34], [35]) is seen in those with longstanding infection. Indeed, the finding that chronic filarial infection is associated with increased numbers of memory but decreased numbers of effector T cells [36] may support the idea of activation and T cell survival being tightly regulated through pro- and anti-apoptotic mechanisms.

Many of the genes differentially expressed by unstimulated T cells of endemic patients have known regulatory and/or inhibitory roles in immune and inflammatory responses. Such molecules included TIMP2, a suppressor of endothelial cell proliferation (a clinical hallmark of filarial infection), the transcription factor IRF2, a competitive inhibitor of IRF1 mediated transcription of IFN-α and β and a factor in the upregulation of FasL [37], and the transcriptional activator and repressor MAF. The increase in MAF, a molecule that plays a role in increased T cell apoptosis as well as in the production of IL-4 and IL-10 (a prominent regulatory cytokine in filarial infection [19]), but inhibits production of IFN-γ and IL-12 [38], supports previous findings of an increased production of IL-4 IL-13 and IL-10 in microfilaremic loiasis patients [39]. Moreover, the interference of IRF2 and MAF with the IFNs strongly suggests an anti-inflammatory role for these molecules that, at the very least, impairs Th1 differentiation in chronically infected patients.

In addition to these regulatory molecules, the receptor for prostaglandin D2 (PTGDR) was upregulated in the unstimulated CD8^+^ cells of endemic patients in comparison to expatriates. This receptor-ligand interaction decreases the migration of Langerhans' cells in the skin [40], the cytotoxicity of NK cells [41], and the expression of both IFN-γ and IL-2 [42], all of which further serves to downregulate both the innate and adaptive immune responses. Moreover, CD8^+^ cells also overexpressed the chemokine ligand CCL4, the secretion of which mediates CD8^+^ T regulatory cells to suppress T cell responses [43]. Taken together, the increased expression of all of these molecules in endemic patients may reflect the lack of clinical symptoms [1], [2] and parasite-specific *in vitro* T cell responses [1], [12] frequently observed in chronically infected patients with filarial infections and suggest possible mechanisms for the regulation of inflammatory activity.

To examine the larger relationships between genes that were differentially expressed by either patient group GSEA was used. Interestingly, those pathways associated with CD8^+^ T cells were the most significant in the unstimulated cells of both patient groups (Figure S2). In endemic patients the IL-5 and eosinophil pathways [44] were significantly enriched in CD8^+^ T cells as well as CD4^+^ T cells. Two highly enriched pathways in endemic patients were associated with T cell-B cell interactions, the ASBCell Pathway (involved in Ag dependent B cell activation) and the BBCell pathway (involved in the induction of apoptosis in Fas-expressing inactive B cells), suggesting a possible role for T cells in the Ag-induced activation and cell death of B cells in filarial infection.

The CD8^+^ T cell pathways identified in the expatriates were related most often to metabolic and cell maintenance functions and included the PAC1 receptor pathway, associated with the activation of adenylyl cyclase and phospholipase C, and the WNT Ca2 cyclic GMP pathway. However, expatriate T cells also demonstrated an enrichment of those pathways involved in killing (granulocyte cell survival and complement/coagulation) as well as those associated with allergic functions (adrenergic pathway). The enhancement of these latter pathways, might serve to explain the augmented pathology associated with infection in expatriates with Loa infection [5], [45] seen to a much lesser degree in those with long-term infection.

For MfAg stimulated recall responses, one common finding between the patient groups was that, unlike the enrichment of CD8^+^ pathways seen in unstimulated cells, it was the pathways in CD4^+^ cells that were significantly enhanced in response to parasite Ag, not surprising given the HLA-Class 2 restriction of many of the T cell responses [46]. Indeed, although the number of altered genes in response to MfAg was far fewer in expatriates in comparison to endemic patients, those genes that were upregulated were closely tied to pathways of the innate and adaptive inflammatory response (i.e. Notch, and TLR pathways; Figure S4). This increase in inflammatory pathways was further supported by the upregulation of molecules associated with NFKB activation and calmodulin-mediated responses (Figure 6 and data not shown) as well as by a downregulation of the apoptotic DNA fragment pathway (Figure S4).

In endemic patients with long-term infection, the response to MfAg was difficult to synthesize fully. Compared with expatriates, where the recall response to MfAg was clearly of an inflammatory nature, the T cell response to MfAg by endemic patients appeared to be balanced between activation and regulation of the immune response. This balance was perhaps most clearly seen in the transcriptional regulation of molecules associated with cell death and apoptosis, similar to the findings in unstimulated cells. The downregulation of the pro-apototic BAD pathway (Figure S3) and the molecule RTN-4, an inhibitor of the anti-apoptotic factors Bcl-2 and Bcl-XL [47], in addition to the upregulation of DHCR24 (an inhibitor of caspase-3; [48]) would counter mechanisms designed to increase cell death, including the upregulation of CXCR4 (Figure 6B and Figure S3), a mediator of CD95-independent cell death in CD4^+^ cells [49], and the downregulation of the receptor and pathways for IGF1, a potent proliferative and anti-apoptotic signaling system (Figures 7 and Figure S3).

Numerous other examples of the opposing nature of certain functions associated with differentially regulated genes in endemic patients were also in evidence. Although several regulatory molecules were upregulated in the unstimulated cells of endemic patients, other such molecules as RARRES3, a retinoic acid family member that functions as a negative regulator of cell proliferation, as well as NFKB1A and PTEN, suppressors of the NFkB and AKT complexes respectively, were all downregulated in MfAg driven cells (Figure 7). Furthermore, several molecules associated with activation (CD69 and and CD247; Figure 6B) and chemokine functions (CCL3 [MIP1γ, also found in expatriate T cells], CCL22 [a chemotactic molecule for chronically activated but not resting T cells]), as well as the canonical pathway for CCR5 were upregulated. Of particular interest is the interaction of CCR5 with its ligand CCL5 which induces the release of histamine from basophils and activates eosinophils, two common features associated with filarial infection [50].

Several other multi-functional complexes upregulated in the parasite Ag-driven cells of endemic patients may offer clues to mechanisms underlying the depressed T cell responses typically seen in these patients. Ubiquitin and its associated molecules (Figure 6B) function in a wide variety of cellular processes including Ag presentation and apoptosis and, through a post-translational modification, mark proteins for degradation. Recently, GRAIL (the E3-ubiquitin ligase gene related to anergy in lymphocytes) was shown to be responsible for the Th2 hyporesponsiveness in a mouse model of chronic schistosomiasis [51], a finding suggested by data from human filarial infections [12], [20], [52]. In addition, several genes linked to the histone family of molecules were also upregulated, including the gene for histone h3. This molecule, through an ERK dependent mechanism, allows the binding of transcription factors to the IL-10 promoter and subsequent expression of the IL-10 gene, which, as mentioned previously, is a prominent regulatory molecule associated with chronic filarial infection [53], [54]. The additional finding of increased expression of molecules associated with insulin has parallels in two recent studies in which: 1) activation of the IL-4/Stat6 pathway, important in immunity to helminths [55], [56], has been shown to increase insulin action during helminth infection [57], and 2) there was a relationship between eosinophil production of IL-4, alternatively activated macrophages in adipose tissue, and enhanced glucose tolerance in a mouse model of *Nippostrongylus brasiliensis* infection [58].

Finally, when the CD4^+^ T cell responses to the nonparasite Ag SLO were analyzed, an upregulation of several molecules associated with activation and inflammation (chemokines, cytokines, others) was seen in the T cells of both endemic and expatriate patients. Indeed, since many studies have demonstrated the similarity in cytokine and proliferative responses to nonparasite Ags even between filarial-infected and -uninfected individuals [59], the overlap of gene expression between patient groups in the CD4^+^ T cell microarray data would be expected. These similarities were not seen in the SLO response in CD8^+^ T cells, responses that were extremely different between the two groups. Of particular note was the observation that Stat-1, important for IFN signaling, was not upregulated in the CD8^+^ cells of endemic patients as it was in CD4^+^ cells as well as in both T cell types of expatriates. With the recent findings that helminth-infected individuals have altered responses to *Mycobacterium tuberculosis* [60], malaria [54], HIV [61], and even to vaccines [62], [63], [64], it may be that CD8^+^ T cells play a larger role in the global modulation of the immune system seen in patients with chronic helminth infection.

The present study thus demonstrates that the clinical and immunological differences previously observed between endemic and expatriate patients can be demonstrated at the transcriptional level in unstimulated (*ex vivo*) cells, during early recall responses to parasite Ag, and even to nonparasite Ag. Indeed, the transcriptional differences between the two groups of filarial-infected patients reflect many of the differences that are seen between acute and chronic viral infection [65]. While the microarray findings in this study by no means constitute the final analysis of the differences between patients with long-standing infection and those with more recently acquired infection, they do suggest several mechanisms that warrant further investigation. It must be argued, therefore, that chronicity (and possibly *in utero* or neonatal exposure to filarial antigens) helps define the disparities between these two groups of patients. Further characterization of the differences seen between long-term and newly acquired infection could help to define the natural progression of filarial infection and the responses that underlie this progression.

## Supporting Information

Figure S1 **GSEA analysis of canonical pathways in unstimulated CD4^+^ cells.** Gene Set Enrichment Analyis (GSEA) of canonical biological pathways in the unstimulated CD4^+^ T cells of endemic (in red) and expatriate (in blue) filarial-infected patients. The heat map represents the values of differentially expressed genes in the 4 media samples (END = endemic; EXP = expatriate) of each patient. Genes in brown represent those upregulated in one patient group with respect to the other while genes in blue are downregulated. Enrichment of biological functions was determined by clustering analysis showing significant differential expression in the CD4^+^ T cells of endemic (top right panel) and expatriate (bottom right panel) patients. Each bar in the plot represents the percent enrichment of a particular pathway (top axis) and an asterisk represents a significant corresponding p-value (FDR<0.1) for the pathway (bottom axis).
(TIF)

Figure S2 **GSEA analysis of canonical pathways in unstimulated CD8^+^ cells.** Gene Set Enrichment Analyis (GSEA) of canonical biological pathways in the unstimulated CD8^+^ T cells of endemic (in red) and expatriate (in blue) filarial-infected patients. The heat map represents the values of differentially expressed genes in the 4 media samples (END = endemic; EXP = expatriate) of each patient. Genes in brown represent those upregulated in one patient group with respect to the other while genes in blue are downregulated. Enrichment of biological functions was determined by clustering analysis showing significant differential expression in the CD8^+^ T cells of endemic (top right panel) and expatriate (bottom right panel) patients. Each bar in the plot represents the percent enrichment of a particular pathway (top axis) and an asterisk represents a significant corresponding p-value (FDR<0.1) for the pathway (bottom axis).
(TIF)

Figure S3 **GSEA analysis of canonical pathways in MfAg-driven CD4^+^ endemic cells.** Gene Set Enrichment Analyis (GSEA) of canonical biological pathways in the microfilarial Ag (MfAg) stimulated CD4^+^ T cells of endemic filarial-infected patients. The heat map represents the values of differentially expressed genes in each of the 3 patients to MfAg (on the right) and to the corresponding media values (on the left). Genes in brown represent those upregulated to MfAg with respect to media while genes in blue are downregulated. Enrichment of biological functions was determined by clustering analysis showing significant differential expression in the CD4^+^ T cells for upregulated genes (green bars, top right panel) and downregulated genes (blue and purple bars, bottom right panel). Each bar in the plot represents the percent enrichment of a particular pathway (top axis) and an asterisk represents a significant corresponding p-value (FDR<0.1) for the pathway (bottom axis).
(TIF)

Figure S4 **GSEA analysis of canonical pathways in MfAg-driven CD4^+^ expatriate cells.** Gene Set Enrichment Analyis (GSEA) of canonical biological pathways in the microfilarial Ag (MfAg) stimulated CD4^+^ T cells of expatriate filarial-infected patients. The heat map represents the values of differentially expressed genes in each of the 3 patients to MfAg (on the right) and to the corresponding media values (on the left). Genes in brown represent those upregulated to MfAg with respect to media while genes in blue are downregulated. Enrichment of biological functions was determined by clustering analysis showing significant differential expression in the CD4^+^ T cells for upregulated genes (green bars, top right panel) and downregulated genes (blue bars, bottom right panel). Each bar in the plot represents the percent enrichment of a particular pathway (top axis) and an asterisk represents a significant corresponding p-value (FDR<0.1) for the pathway (bottom axis).
(TIF)

Table S1 **Quantitative RT-PCR and microarray values.** Comparison of quantitative RT-PCR (Taqman™) and microarray expression values in CD4^+^ and CD8^+^ unstimulated T cells. The data correspond to graphs illustrated in Figure 2 and represent the average values for the three individuals within each patient group.
(DOC)
