# Supplementary figures and images for: Regulation of Global Gene Expression in Human Loa loa Infection Is a Function of Chronicity
Source: PLoS Negl Trop Dis. 2012 Feb 28;6(2):e1527. doi: 10.1371/journal.pntd.0001527 (PMC3289604; doi:10.1371/journal.pntd.0001527)

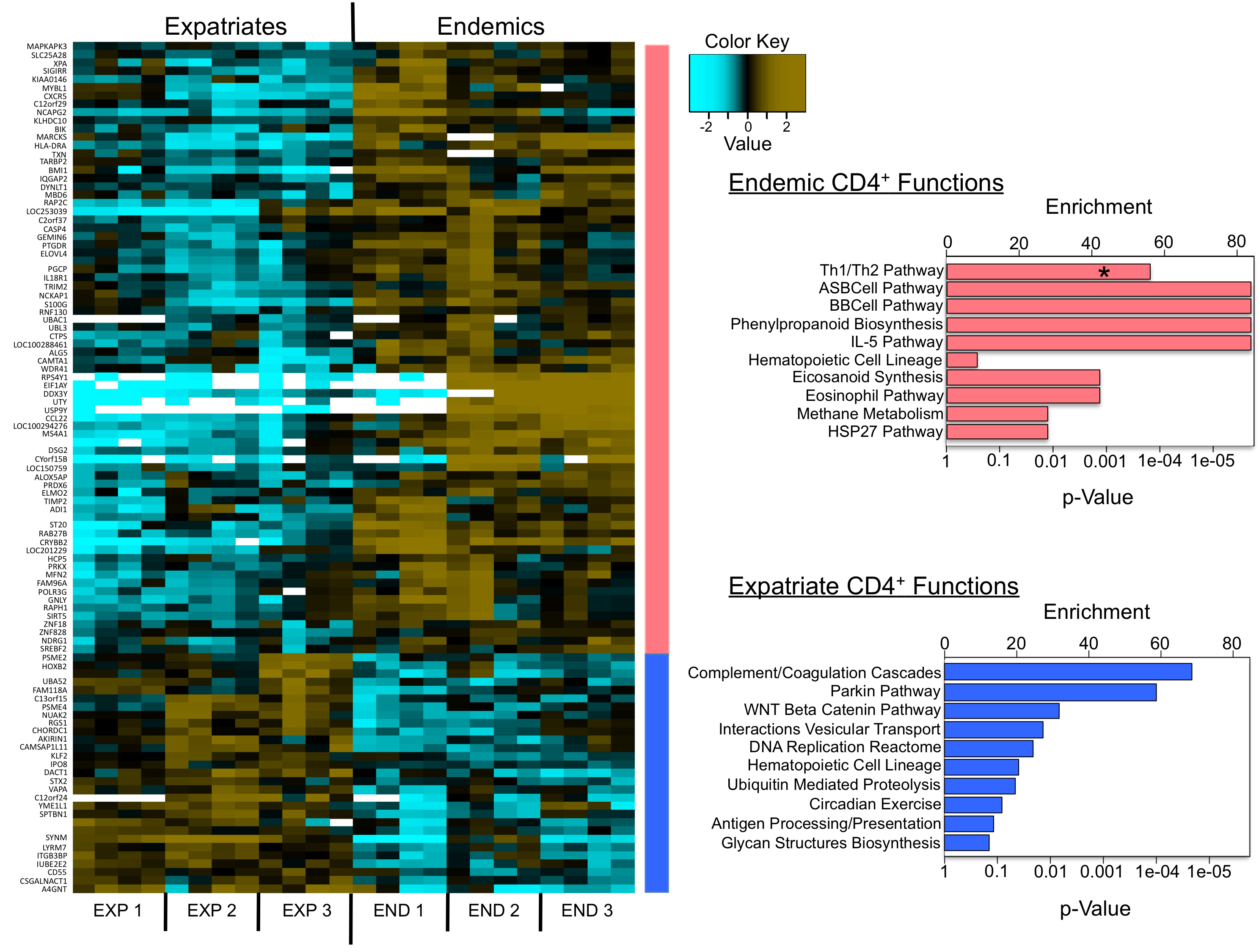

Supplement: Figure S1 — GSEA analysis of canonical pathways in unstimulated CD4+ cells. Gene Set Enrichment Analyis (GSEA) of canonical biological pathways in the unstimulated CD4+ T cells of endemic (in red) and expatriate (in blue) filarial-infected patients. The heat map represents the values of differentially expressed genes in the 4 media samples (END = endemic; EXP = expatriate) of each patient. Genes in brown represent those upregulated in one patient group with respect to the other while genes in blue are downregulated. Enrichment of biological functions was determined by clustering analysis showing significant differential expression in the CD4+ T cells of endemic (top right panel) and expatriate (bottom right panel) patients. Each bar in the plot represents the percent enrichment of a particular pathway (top axis) and an asterisk represents a significant corresponding p-value (FDR<0.1) for the pathway (bottom axis). (TIF) [file pntd.0001527.s001.tif]

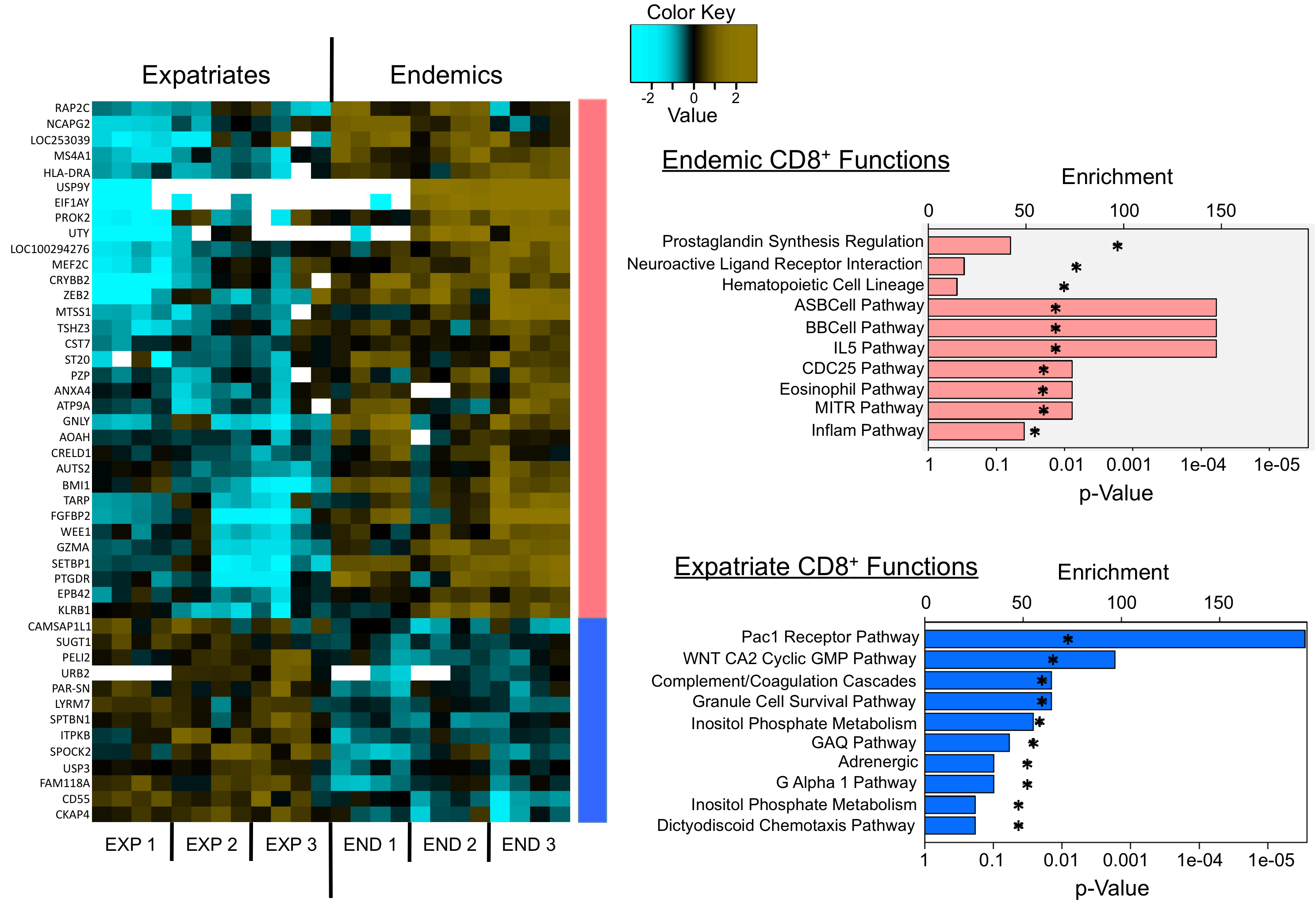

Supplement: Figure S2 — GSEA analysis of canonical pathways in unstimulated CD8+ cells. Gene Set Enrichment Analyis (GSEA) of canonical biological pathways in the unstimulated CD8+ T cells of endemic (in red) and expatriate (in blue) filarial-infected patients. The heat map represents the values of differentially expressed genes in the 4 media samples (END = endemic; EXP = expatriate) of each patient. Genes in brown represent those upregulated in one patient group with respect to the other while genes in blue are downregulated. Enrichment of biological functions was determined by clustering analysis showing significant differential expression in the CD8+ T cells of endemic (top right panel) and expatriate (bottom right panel) patients. Each bar in the plot represents the percent enrichment of a particular pathway (top axis) and an asterisk represents a significant corresponding p-value (FDR<0.1) for the pathway (bottom axis). (TIF) [file pntd.0001527.s002.tif]

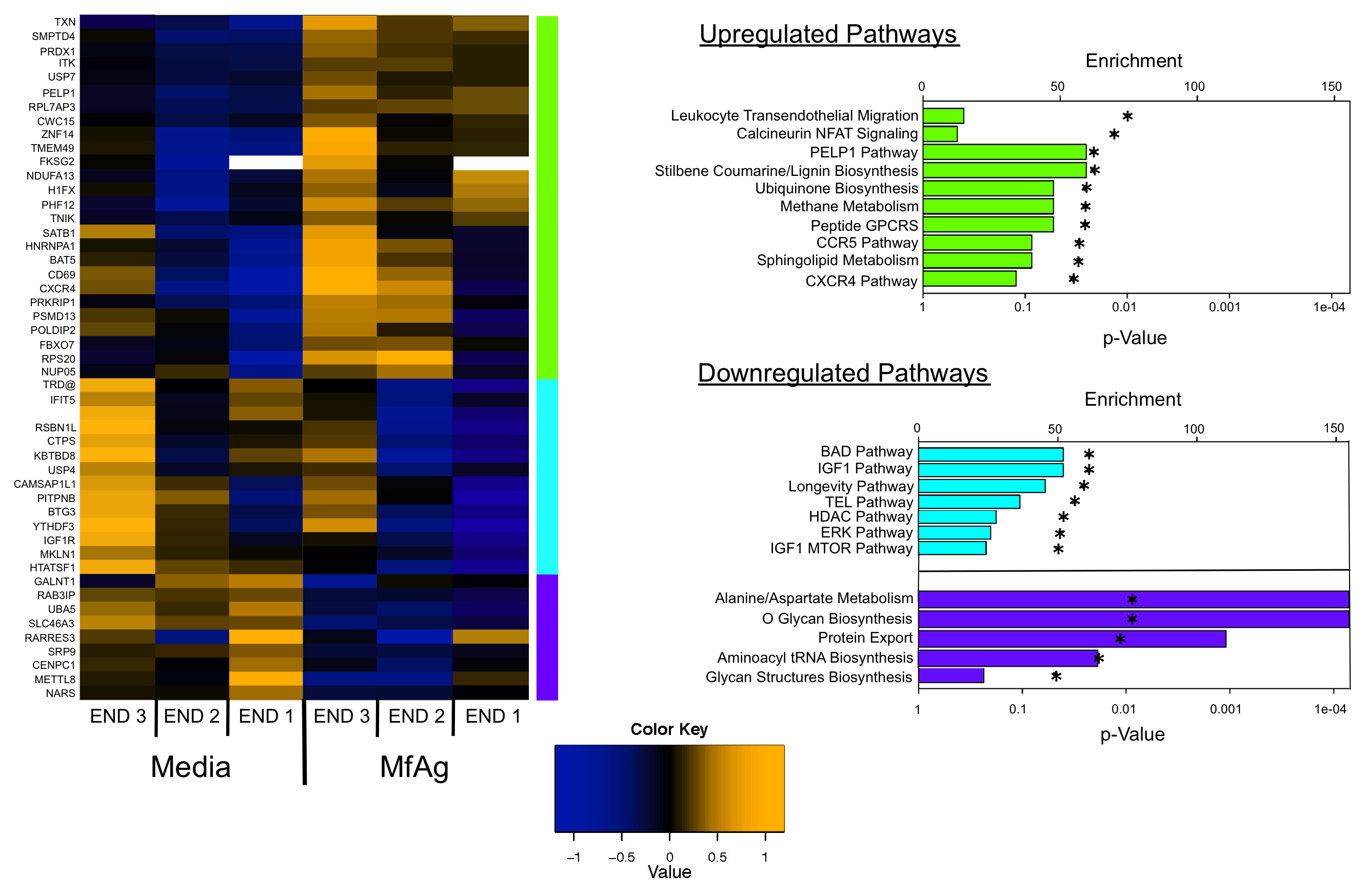

Supplement: Figure S3 — GSEA analysis of canonical pathways in MfAg-driven CD4+ endemic cells. Gene Set Enrichment Analyis (GSEA) of canonical biological pathways in the microfilarial Ag (MfAg) stimulated CD4+ T cells of endemic filarial-infected patients. The heat map represents the values of differentially expressed genes in each of the 3 patients to MfAg (on the right) and to the corresponding media values (on the left). Genes in brown represent those upregulated to MfAg with respect to media while genes in blue are downregulated. Enrichment of biological functions was determined by clustering analysis showing significant differential expression in the CD4+ T cells for upregulated genes (green bars, top right panel) and downregulated genes (blue and purple bars, bottom right panel). Each bar in the plot represents the percent enrichment of a particular pathway (top axis) and an asterisk represents a significant corresponding p-value (FDR<0.1) for the pathway (bottom axis). (TIF) [file pntd.0001527.s003.tif]

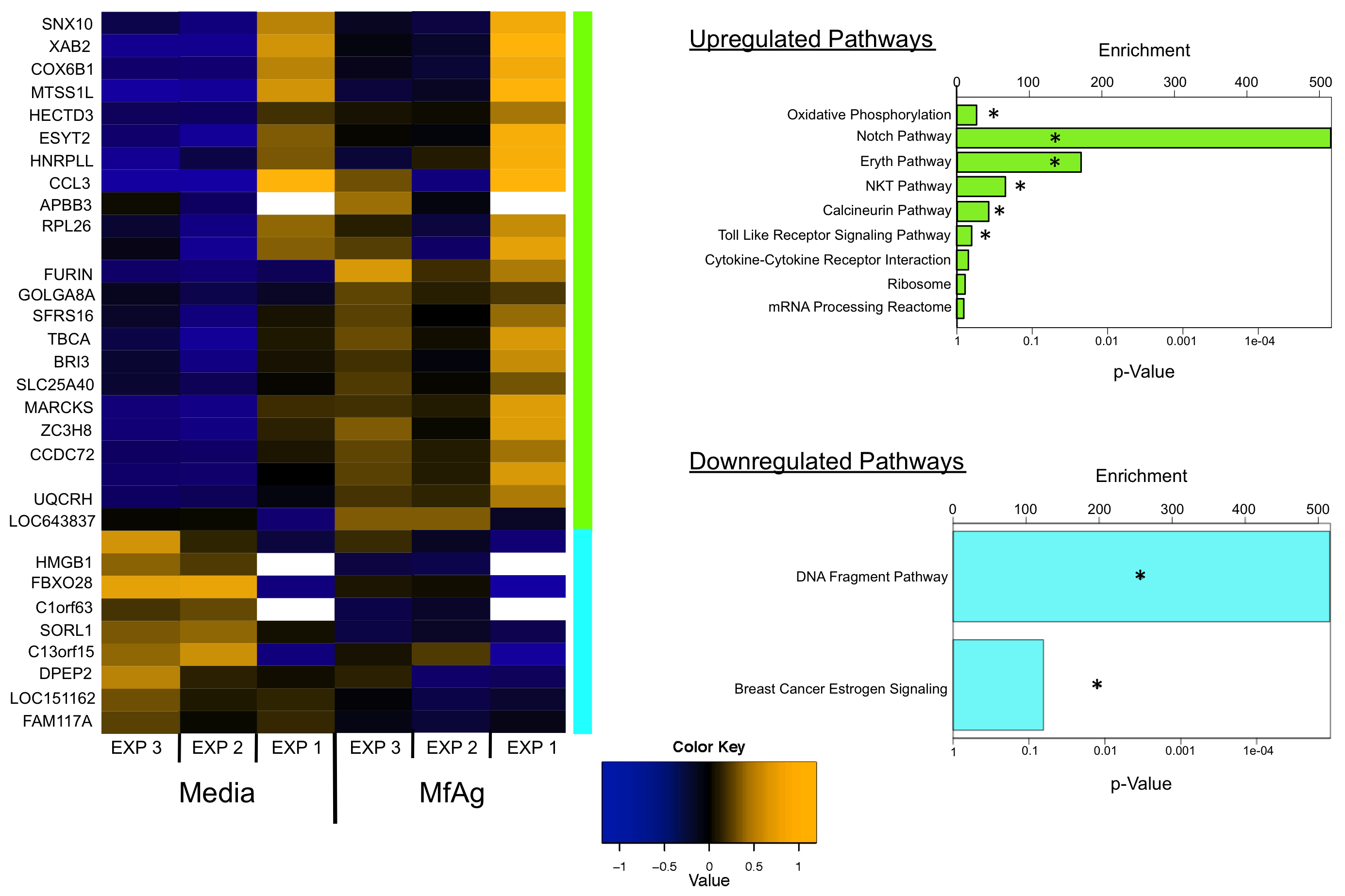

Supplement: Figure S4 — GSEA analysis of canonical pathways in MfAg-driven CD4+ expatriate cells. Gene Set Enrichment Analyis (GSEA) of canonical biological pathways in the microfilarial Ag (MfAg) stimulated CD4+ T cells of expatriate filarial-infected patients. The heat map represents the values of differentially expressed genes in each of the 3 patients to MfAg (on the right) and to the corresponding media values (on the left). Genes in brown represent those upregulated to MfAg with respect to media while genes in blue are downregulated. Enrichment of biological functions was determined by clustering analysis showing significant differential expression in the CD4+ T cells for upregulated genes (green bars, top right panel) and downregulated genes (blue bars, bottom right panel). Each bar in the plot represents the percent enrichment of a particular pathway (top axis) and an asterisk represents a significant corresponding p-value (FDR<0.1) for the pathway (bottom axis). (TIF) [file pntd.0001527.s004.tif]
