# Supplementary material for: Regulation of Global Gene Expression in Human Loa loa Infection Is a Function of Chronicity
Source: PLoS Negl Trop Dis. 2012 Feb 28;6(2):e1527. doi: 10.1371/journal.pntd.0001527 (PMC3289604; doi:10.1371/journal.pntd.0001527)
Supplement: Table S1 — Quantitative RT-PCR and microarray values. Comparison of quantitative RT-PCR (Taqman™) and microarray expression values in CD4+ and CD8+ unstimulated T cells. The data correspond to graphs illustrated in Figure 2 and represent the average values for the three individuals within each patient group. (DOC) [file pntd.0001527.s005.doc]

Table S1. Comparison of Taqman and Microarray Group Values in

Endemic and Expatriate Loiaisis Patients*

| Taqman Group Values | | | Microarray Group Values | |
| --- | --- | --- | --- | --- |
|  | Endemics | Expatriates | Endemics | Expatriates |
| **CD4+ Cells** | | | | |
| PTGDR | 32.3 | 24.4 | 208.4 | 104.6 |
| CCL4 | 136.5 | 84.6 | 635.1 | 242.3 |
| DIABLO | 187.0 | 185.6 | 1056.1 | 417.3 |
| BIRC3 | 612.8 | 548.4 | 3707.0 | 1810.2 |
| CCL3 | 43.4 | 36.4 | 460.4 | 171.4 |
| ELK1 | 159.3 | 146.5 | 1775.6 | 837.8 |
| HLA-DRA | 153.6 | 78.0 | 350.6 | 98.9 |
| MS4A1 | 12.7 | 11.0 | 264.8 | 76.9 |
| MEF2C | 39.0 | 27.7 | 134.1 | 81.1 |
| MYBL1 | 60.1 | 43.4 | 2383.8 | 901.7 |
| **CD8+ Cells** | | | | |
| PTGDR | 58.0 | 37.8 | 1587.5 | 472.6 |
| CCL4 | 449.0 | 265.3 | 6529.8 | 2200.3 |
| DIABLO | 212.9 | 216.5 | 763.4 | 440.0 |
| KLRB1 | 324.1 | 154.1 | 3051.8 | 1474.9 |
| HLA-DRA | 343.6 | 125.5 | 482.9 | 175.8 |
| MS4A1 | 18.8 | 12.9 | 541.4 | 174.0 |
| FGFBP2 | 61.4 | 35.9 | 2280.4 | 603.3 |

* Data corresponds to the graphic data in Figure 2. Final values

were obtained by averaging the values for the 3 patients within

each patient group.
